# Consumptive and Non-Consumptive Uses of Water Beetles (*Aquatic coleopterans*) in Sub-Saharan Traditional Rituals

**DOI:** 10.3390/insects14100795

**Published:** 2023-09-29

**Authors:** Lucky Nhlanhla Mnisi, Nompumelelo Zondi, Innocent Pikirayi

**Affiliations:** 1 Department of African Languages, University of Pretoria, Hatfield 0028, South Africa; mpume.zondi@up.ac.za; 2 Department of Anthropology, Archaeology and Development Studies, University of Pretoria, Hatfield 0028, South Africa; innocent.pikirayi@up.ac.za

**Keywords:** water beetles, resource use, biotic resources, human–animal scholarship, ethnomedicine, entomophagy, magico-religious

## Abstract

**Simple Summary:**

Despite the considerable amount of research conducted on the roles of water beetles in African cultural rituals, their long-term viability for use in these rituals has not been sufficiently investigated. This study was, therefore, conducted in an effort to address this lacuna in human–animal scholarship. We examined the traditional rituals through a consumptive versus non-consumptive lens. Although the notions of consumptive and non-consumptive resource use have been widely explored in other fields such as water resource management and the study of mammals and other vertebrate species (in ecotourism), their application to human–aquatic insect interactions is currently sparse. Notwithstanding the need for further research in this area, our assessment was inexhaustive in establishing conclusive differentiations about the consumptive or non-consumptive nature of the majority of the rituals. Thus, most of the classifications were hypothetical. Certain aspects of the challenges encountered could be attributed to the inexplicit nature of consumptive and non-consumptive concepts when applied to human–animal interactions and use. As a result, our study shows that the application of categorization methods specifically tailored for abiotic resources may encounter limitations when applied to biotic resources.

**Abstract:**

The use of wild animals in customary rituals and as a sustenance resource is a longstanding tradition within sub-Saharan Africa. The emergence of commercial trade, has, however, created unattainable demands and has led to the overexploitation of animals. These demands are threatening the conservation of animal species exploited in this trade. Comparatively little research effort has been dedicated to invertebrate species, and, specifically, their non-commercial uses. We explored the uses of water beetles in traditional rituals. We investigate the extent to which each of the non-commercial uses of water beetles exhibits consumptive and non-consumptive use features. The concepts are contested as their application for describing human–animal interactions has been challenged because of insufficient physiological and conservation data on the implications for animals of such interactions. The inadequacy of the available data pertaining to the use of animal resources was particularly pronounced. Most research efforts are skewed towards vertebrates at the expense of invertebrates. Regardless, the study shows that most non-commercial exploitation and uses of water beetles were mainly non-destructive and, if consumptive, the uses could be described as mainly non-lethal consumptive or sub-lethal consumptive. Rituals that could be described as lethal-consumptive comprised a smaller fraction of the uses of water beetles.

## 1. Introduction

The interaction between humans and natural ecosystems is intricate. Human survival is heavily reliant on natural ecosystems for a wide range of goods and services, including the provisioning of material goods such as food, regulation (e.g., biological control), cultural (provision of non-material benefits such as recreation and leisure, spiritual, symbolic value, and aesthetics), and supporting (primary production, pollination, decomposition, soil formation) [1,2]. As humanity continues to derive and enjoy the benefits provided by the natural ecosystems, it is essential to maintain awareness of the fragile balance between resource stocks, inflows (reproduction or replenishment rates), and outflows (harvesting or depletion rates). User consideration is a significant aspect that needs to be taken into account given that the natural ecosystem’s capacity to provide these benefits is not without limits [3]. Thus, the exploitation of natural ecosystems’ resources requires rigorous examination and accountability to prevent depleting their reserves and interfering with nature’s limited capacity for restoration.

Regrettably, conservation statistics depict a grim picture in this regard. It is estimated that over 28% of animal species listed on the International Union for Conservation of Nature (IUCN) Red List face imminent risk of extinction [4]. Habitat destruction, invasive species, overexploitation, illegal wildlife trade, pollution (light, noise, and chemical), and climate change are the most pressing issues facing biodiversity conservation today, generating resultant threats of extinction [5]. A desktop assessment of the IUCN Red List database of threatened species (and environmental threats responsible for endangered conservation status) placed overexploitation as the second most dominant threat [5].

Overexploitation occurs when the harvesting rate of a resource is greater than its replenishment rate [6]. Humans exploit non-human animals mainly for subsistence and profit and to a lesser extent for recreational purposes [7]. Conservation biologists and resource-use economists have raised biodiversity concerns over the increased exploitation of animal species, a rate exacerbated by the soaring global demands for the use of animal species in traditional medicines (e.g., zootherapy, pharmacology, and for practising spiritual beliefs) [8,9,10,11,12,13]. While the use of animals in traditional medicine is considered one of the emerging threats to biodiversity, we note that the use of animals in traditional or folk medicines for treating ailments and fulfilling magico-religious and spiritual requirements is not new in Africa [12,14]. Despite the historical reliance on animals for medical purposes, strong regulations must be adopted to prevent the overexploitation of highly sought-after species [15].

Insects and other arthropods, for example, have long been used in human diets and traditional medicines. The reliance on insects for these purposes has been shown to be widespread not just in Africa, but also in East Asia and South America [16]. According to Matandirotya et al. [17], a variety of arthropods, including ants, termites, bugs, grasshoppers, wild silkworms, beetles, crickets, caterpillars, bees, and locusts, are recognised as prominent and commonly consumed throughout Africa. In addition, the literature indicates that these organisms are consumed not solely because of their nutritional benefits but also because they are thought to possess properties necessary for the treatment of certain ailments [17,18].

Water beetles constitute one of the most dominant groups of macroinvertebrates inhabiting non-marine environments [19,20]. Taxonomic documentation suggests that over 13,000 species have been described thus far [20], while biogeographical information indicates that water beetles are found on all continents, except Antarctica [20]. Their dominance transcends beyond their numbers and diversity. Where they are found, water beetles serve numerous ecological functions and provide practical benefits to human societies. For example, water beetles (i) serve as nutrient contributors for higher-trophic-level organisms and maintain a healthy ecosystem by preying on lower-trophic-level species; (ii) are used in biological monitoring of ecosystems (they are used as valuable indicators of water quality and ecosystem health); (iii) are used as fishing baits by fishing communities; and (iv) are used as a biological control of noxious flora and fauna [21,22,23]. Water beetles are also becoming increasingly significant in animal research. They have been incorporated into many scientific explorations like DNA taxonomy, microecology, historical biogeography, sexual selection, conservation biology, and understanding how animals will react to climate change [20].

A similar argument can be made in the field of human–animal scholarship (HAS).

Beyond these attributes, beetles are of salient significance in African indigenous cultures and are an inherent part of the indigenous knowledge system. Van Huis [18] explains how beetles are used, perceived, and experienced in daily life across sub-Saharan Africa (SSA). Using a sample of 300 respondents from 27 countries in the region, and comparing the findings with those in the scientific literature, the author shows that many beetle species are consumed, both as adults and larvae. They are also used in a variety of ways such as stimulating breast growth, as an aphrodisiac, and for treating some sexually transmitted diseases.

In addition to the detailed contribution by van Huis, the traditional use of water beetles in African ceremonies has received considerable attention within the fields of HAS and ethnomedicine. Kutalek and Kassa [24], for example, probed the possible medicinal uses of water beetles in East and Southern Africa; Refs. [24,25,26] investigated the contribution of water beetles to human diets. These surveys have provided essential insights into how these insects were and are still used in everyday life by various cultures across the subcontinent. However, the literature on the long-term viability of traditional water beetle use in SSA remains patchy.

Natural resource use of biotic and abiotic resources may be classified as either non-consumptive or consumptive [27,28,29,30,31]. Non-consumptive use of a resource is characterised by non-interventive, metaphoric/symbolic, or intangible uses [28]. Typical examples of non-consumptive uses include viewing or photographing. Although mortality is often used as the major indicator for consumption, it is worth noting that additional (non-lethal) consumption indicators have been highlighted in the literature [32]. These include direct extraction, the removal or withdrawal of all or part of the natural resource from its origin, permanent alteration of the resource [30], and interactions resulting in the removal of parts of the organism and/or parts of its derivatives (e.g., the extraction of venom and its related substances from the animal) [27].

In the classification of natural resource usage, the two terms (consumptive versus non-consumptive) have been used extensively yet indiscriminately. Tremblay [33] highlighted in a review that the origins of the terminologies cannot be completely established; nonetheless, the initial applications of the terminologies can be linked to water resource management. The use of these concepts outside of water resources, or more precisely, for categorising the various uses of animal resources, is still contentious. Despite these debates, these concepts have been widely but disproportionately used in the classification of human–animal interactions (particularly vertebrates), for example, in relation to ecotourism and sustainable tourism management (see, for example, Thomsen et al. [34]), and by ecologists in describing predator–prey interactions (see, for example, [35,36]) and, to a lesser extent, human–macroinvertebrate interactions.

This study examines the long-term sustainability of water beetle use in traditional SSA rituals. In particular, we explore uses and interactions that are untainted by the emergence of commercial trade and the export of animals for profit. First, we investigate how water beetle exploitation for the harvester’s traditional purposes (personal use) takes place in SSA. Second, we explore how each of the uses embodies components of consumptive and non-consumptive interactions. Thirdly, we consider how well each ritual fits into the consumptive versus non-consumptive dichotomy and whether the emphasis on categorisation necessitates additional criteria that extend beyond the dichotomy.

## 2. Materials and Methods

### 2.1. Data Sources

The data utilised in this study were obtained from a combination of secondary and primary sources. The collection of secondary data involved multiple searches on Google Scholar for relevant literature sources, using key search terms and phrases related to water beetles and traditional uses in sub-Saharan Africa. To supplement the word searches, we performed a citation search, which entailed examining the bibliographies of the downloaded articles and identifying suitable literature sources to add to our pool of data sources. To ensure our findings were specific to traditional ritualistic practices involving water beetles in sub-Saharan African countries, we excluded material reporting on uses and interactions involving terrestrial beetles and those indicating use beyond the spatial extent of this region. Our final collection of secondary data sources consisted of data derived from peer-reviewed studies and dissertations.

The primary data collection was carried out through focus group discussions (FGDs) conducted in the KwaZulu-Natal province of South Africa. The identification and recruitment of participants for the FGDs were conducted via a method described as “nomination” [37]. In carrying out this process, the researchers engaged with individuals and leaders of community organisations, providing them with an overview of the study and detailing the specific characteristics of the participants sought. These individuals then assisted the research team in creating a cohort of around eight volunteers for each group.

Highly sought were participants who demonstrated a willingness to participate in the research, were accessible during the scheduled days, times, and locations for the FGDs, and possessed knowledge or experience relevant to the subject matter. Additionally, priority was given to individuals who were brought up in rural regions or presently inhabit rural areas. The rationale for highlighting the rural characteristics of the participants emerged from the recognition that rural populations play a significant role in preserving and safeguarding traditional indigenous knowledge, as previously noted by [38].

In light of the fact that certain rituals documented in the literature were gender specific, we ensured that our groups comprised both men and women. The inclusion of both gender perspectives was a key consideration for achieving a representative sample and for drawing broader insights pertaining to the use of water beetles in traditional rituals. The final pool of participants consisted of 25 individuals residing in the villages of Rosetta in the KZN midlands, Kwandebeqheke, and Zwelibomvu. Among these participants, there were fifteen adult women, accounting for 60% of the total, and ten adult males, making up 40% of the total.

In order to establish a common frame of reference, the research team supplied the participants with visual aids depicting various families of water beetles. The aids were accompanied by brief explanations of the structure, behaviour, habitat, and colour, as well as isiZulu names of the beetles where applicable. The visual aids consisted of A4-sized images of water beetles, along with their respective English and IsiZulu names. Furthermore, we provided field guidebooks on macroinvertebrates that were secured with crocodile clips to indicate specific pages featuring water beetles.

The establishment of a shared frame of reference was critical in this study, as evidenced by the findings of Kutalek and Kassa [24], who noted that water beetle families were prone to confusion in folk taxonomy. On a similar line, ref. [38] shed light on instances of insect misidentification within the isiZulu folk taxonomy. To prevent the occurrence of such errors, we decided to ensure a common point of reference was incorporated at the beginning of our FGDs.

Each conversation commenced with a comprehensive verbal introduction to the research, encompassing information regarding the objectives of the discussions, the responsibilities of the research team, and the rights of the participants, which encompassed safeguarding their anonymity. Detailed records of the responses and discussions were documented. The discussions were structured through five distinct themes, which are as follows:

- List of traditional rituals performed with water beetle;
- Information related to how the beetles are captured;
- Where the rituals are performed;
- How the beetles are used in the fulfilment of the rituals;
- What happens to the beetles after they are used.

The FGDs gave us an opportunity to obtain insights into local knowledge of water beetles, the degree and nature of any interactions, and their cultural importance. The literature research on the subject provided additional information about the traditional uses of water beetles as well as their significance for the subcontinent, which supplemented the data obtained through the FGDs. The combination of these two methodologies allowed for a thorough knowledge assemblage of how humans engage with these organisms and why they are culturally significant throughout sub-Saharan Africa. The combined use of FGDs and literature searches included themes related to consumptive and non-consumptive uses of water beetles, methods of capturing the beetles, places where rituals are practised, how the rituals are performed, and whether after use the beetles are released alive or dead. Additionally, we explored how each of the interactions affects the beetles’ behaviour and physiology and how these impacts inform the classification of the uses into consumptive, non-consumptive (non-lethal), and non-lethal consumptive. And, lastly, we navigated the difficulties experienced in categorising the rituals into either consumptive or non-consumptive uses.

### 2.2. Ethical Considerations and Clearance

The researchers adhered to all the required protocols for conducting research involving human subjects. Prior to performing fieldwork, we sought ethical clearance from the University of Pretoria Research Ethics Committee. Furthermore, before participating, participants were given adequate details about their rights, as well as the protection of their privacy, and informed that their participation in the study would be voluntary and without remuneration. Although no financial compensation was provided to the participants, we took the initiative to arrange refreshments for them. Participants endorsed their consent to participate by signing the consent forms.

### 2.3. Predetermined Classification Criteria for the Uses of Water Beetles in Traditional SSA Rituals

We classified the uses of the water beetles based on the degree of human–water beetle interaction and the level of harm that the interaction might potentially have on the water beetle. The classification criteria drew insights from the existing literature related to the traditional uses of water beetles. From these criteria, we established four categories: (i) symbolic and metaphoric use; (ii) intangible use; (iii) uses of water beetles involving contact (non-lethal catch–use–release); and (iv) lethal interaction (catch–prepare–ingest).

#### 2.3.1. Symbolic and Metaphoric Use

For some cultures, water beetles represent potent symbols and metaphors that extend beyond their physical form. Even in the absence of their physical form, water beetles have been acknowledged, respected, and celebrated for their mere existence; see, for example, [18,24,39].

#### 2.3.2. Use of Water Beetles as Biological Indicators of Water Quality (Involving No Contact between Humans and the Beetles)

Using water beetles does not necessarily entail physical contact or capture. This category of use entails visiting the habitat and observing the organisms in their natural environment, as well as drawing inferences about the human potability of the water source; see, for example, [18].

#### 2.3.3. Uses of Water Beetles Involving Contact (Non-Lethal) Catch–Use–Release (Alive)

In this category, we considered uses of water beetles that involve a higher level of interaction with the organisms. It differs from the previous two categories in that it entails contact with the beetles, with the handler performing a catch–use–release cycle. The “use” interphase may be accompanied by the release of the beetle’s derivative (venom or other defence compounds), displacement from its habitat source, a loss of energy, an effect on its mobility, its ability to escape predation, and its search for prey resulting from physical harm.

#### 2.3.4. Lethal Interaction (Catch–Prepare–Ingest)

Catch–prepare–ingest is an extreme use of water beetles, as it entails not only interacting with the beetle’s habitat but also capturing and destroying potentially large numbers of the organisms. This may have a direct effect on the population size of the organisms, with potential long-term consequences for species survival.

## 3. Results

The research yielded a wealth of information. The focus groups conducted in the KwaZulu-Natal province provided comprehensive insights into how water beetles are utilised in a variety of rituals, while the review of the literature provided additional data and context for the study. Our findings suggest that water beetles have extensive utility and relevance to traditional practices in SSA.

The investigation amassed a total of 14 distinct traditional rituals that involve water beetles, three of which were gathered from the KZN focus groups. It is noteworthy that the practices gathered from the FGDs were not exclusive to the KZN province, as all of them were observed to be prevalent or practised elsewhere within SSA. This observation indicates that the use of water beetles for ritualistic intentions is prevalent in SSA, highlighting shared characteristics among belief systems in the subcontinent.

### 3.1. Summary of Responses Gathered from FDGs Regarding Traditional Ceremonies Involving Water Beetles

1. In certain regions of Malawi, it is believed that if a whirligig beetle (Gyrinidae) bites you, you will learn to swim and levitate like it. But for this to occur, a whirligig must be placed on the hand. Then, the whirligig will bite its handler. After being bitten by the insect, the handler returns it to the water.
2. In certain regions of Zimbabwe, girls go swimming with the intention of capturing whirligig beetles and placing them on their nipples so that the insect will bite their breasts in the belief that the bite will cause their breasts to grow larger. The girls would release the beetle back into the water after being bitten. The breasts expand, and the girls believe that the whirligig’s supernatural powers are responsible for their enlargement.
3. People who cannot whistle use the whirligig beetle to improve their whistling abilities. They secure one of the numerous beetles in the river and place it on their tongue. They believe that after being bitten, they will be able to whistle. The handler returns the insect to the water after sustaining a bite from the beetle.

Table 1 shows that most of the water beetle rituals obtained through the FGDs consist either entirely of beetles belonging to the Gyrinidae family, commonly referred to as whirligig beetles, or have maintained a non-specific categorisation, referring only to “water beetles”. All the uses gathered through the FGDs involved catch–use–release, with no mortality resulting from the use of the beetles.

### 3.2. Traditional Uses of Water Beetles Gathered from the Literature

As shown in Table 2, the beetles are generalised as ‘water beetles’, and where specified, beetles belonging to the families Gyrinidae (whirligig beetles) and Dytiscidae (predaceous diving beetles) appear to be the most commonly used beetles in traditional rituals. Additionally, we note that water beetles feature in many traditional rituals and are even eaten as a source of food in many parts of the subcontinent.

In this investigation, traditional rituals were assessed and viewed as interactions. The research revealed a spectrum of interactions between humans and water beetles, ranging from those with no effect to those that may jeopardise biodiversity. We identified four distinct categories: metaphoric/symbolic uses, intangible uses, catch–use–release interactions, and uses leading to lethality.

**Figure 1:**
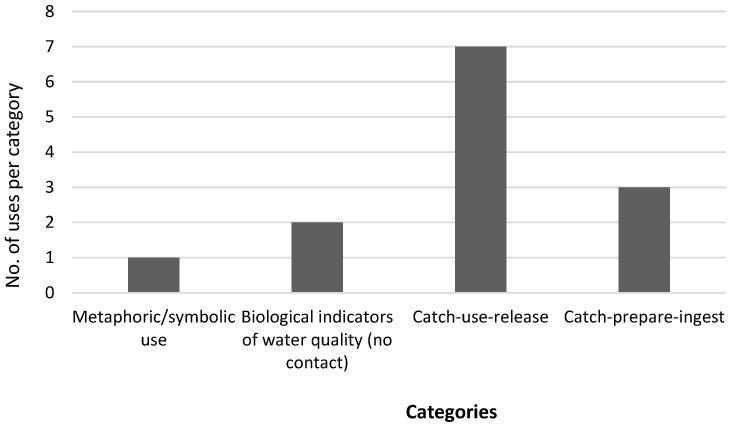
Categories of the uses of the traditional African water beetles gathered from the current study and the literature.

The first group comprises the metaphoric use of water beetles, the second group we described as metaphoric/symbolic or intangible, or non-interventive use of water beetles; while the third group is composed of non-lethal uses that involve catching, using, and releasing the beetles. The last group consists of uses that require the mortality of the beetles, e.g., eating the beetles both as a source of food and for medicinal purposes (Figure 1).

## 4. Discussion

Water beetles have been studied and documented extensively, either independently or in conjunction with other insects. On the downside, however, the sustainability aspect of using water beetles in traditional African rituals is lacking. This study, therefore, considers the ramifications of their use in traditional African rites. We investigated the use of water beetles in traditional SSA rituals, specifically through the lens of a consumptive versus non-consumptive use dichotomy.

This exploration was not devoid of challenges. This was not unexpected given the [33] previous observation that the terms “consumptive” and “non-consumptive” have ambiguous historical roots that are largely tied to water resource management. This raises concerns about their application in the classification of biotic resource use.

### 4.1. Metaphoric or Symbolic Uses of Water Beetles

Water beetles are deeply embedded in African traditional beliefs and rituals. They have been documented to have symbolic meaning in traditional rituals. Gyrinids, for example, have symbolic meaning and significance in sorcery, and dytiscids appear in morality proverbs (see, for example, van Huis [18]). From previous studies, we gathered that the utility of water beetles for the fulfilment of these two rituals neither requires the presence of the beetles nor involves any physical interaction with the beetles ([42], Houlder [39], cited in van Huis [18]).

The swimming behaviour of gyrinids in the context of sorcery is commonly interpreted as representative of a curse. The curse is centred on the unsightly swimming movement and behaviour of gyrinids when disturbed. In a similar vein, scholars have documented the metaphorical representation of dytiscids in Madagascar, as opposed to their literal manifestation ([34], cited in [18]). Both uses require a knowledge of the beetles’ behaviours. The usage of beetles in symbolism and metaphor is limited to recognising the insect and its activities, thereby necessitating neither the actual presence nor disruption of the beetle’s habitat. The classification of this beetle use as non-consumptive is based on its lack of interference with the insect’s environment and absence of physiological impacts.

### 4.2. Biological Indicators of Water Quality

Aquatic organisms, in particular, macroinvertebrates (including water beetles), are the most commonly used biological indicators of water quality and ecosystem health. They feature in monitoring systems of rivers used by ecologists in a process widely known as biological monitoring (biomonitoring) [43]. Similarly, indigenous African communities have relied on macroinvertebrates’ presence and absence as indicators of the prevailing state of the aquatic environment of interest for a very long time. In this category of interactions, we examined the extent to which the use of water beetles (dytiscids) in traditional biomonitoring of water quality, as well as assisting fishing communities in determining if fish are present or absent in a body of water [18], constitutes consumption.

In our investigation, we noted that, in conservation studies, the term intangible has been used interchangeably for non-consumptive uses of natural resources, e.g., [28]. However, the intangibility of animals does not automatically qualify the use or interaction as non-consumptive. Some non-interventive intangible uses, like wildlife and marine life viewing, despite not entailing physical contact, and despite being non-lethal, have been classified as consumptive. Insights from [44,45] indicate that activities described as intangible and non-lethal may in fact have disturbing effects on animals. Such activities and interactions may to some extent entail certain physiological effects arising from the animal’s instinctual risk-avoidance response to threats (Lima and Dill [46], cited in Christiansen and Lusseau [47]). Studies have shown that these disturbances may have far-reaching effects on certain animals, such as interfering with their ability to reproduce and subsequently impacting the population size of the affected species [47]. The full spectrum of actual disturbances on water beetles evoked or impacted by invasions cannot be fully ascertained. This is due, in particular, to insufficient bioassessment data on the implications of interactions between humans and water beetles.

### 4.3. Catch–Use–Release

Uses in this category are by definition non-lethal. From this category, we identified three distinct interactions between humans and water beetles: catch, use, and release. All reported uses of water beetles (gyrinids) collected from the KZN province through the FGDs fall under this category. While the KZN uses and rituals could arguably be considered consumptive, we noted that all three uses are non-lethal, and if the beetles are used and released in situ, they could safely be classified as non-destructive (does not involve the permanent removal of beetles from their habitats or have a direct negative impact on their population size) but not fully non-consumptive.

From the current study and the literature search, we gathered that the handlers employ unsophisticated methods and tools for capturing water beetles. None of the literature indicates the use of sophisticated capture methods or tools specially constructed for capturing multiple beetles. The use of indiscriminative sampling methods like mesh nets and gillnets could be detrimental to biodiversity. Perhaps that is why Dallas [48] classified gillnets as a destructive sampling method. The capturing methods documented in the literature include using hands and sometimes cups and washing basins [24]. Insights from the current study and the literature indicate that all rituals under this category utilise live beetles. It may, thus, be inferred that the beetles are meticulously captured to ensure the preservation of their vitality, thereby enabling those involved to carry out the intended rituals with optimal efficacy.

The handlers believe that the beetles have to bite specific body organs to realise the magical benefits of the beetles. For example, the beetle has to bite the tip of the tongue for unlocking whistling potential; it has to bite the palm or the abdomen (stomach) of the users to improve swimming efficiency; see, for example, [18]. An exception is when the beetle is used in the treatment of dizziness and in water purification. In both cases, users do not require the insects to bite them for the realisation of their magical powers. Ideally, if the ritual is performed in situ, none of the rituals in this category result in mortality, permanent alteration of the handled beetles, or permanent removal from the natural environment.

While aquatic beetles have been reported not to be aggressive to humans [49], note that they do bite when handled. It is natural for an insect to display defensive mechanisms and antipredator strategies when handled. According to [50], aquatic insects rely on a variety of defence traits including escape, mechanical defences, stridulation, and chemical deployment. Other insects use chemicals to deter predation [51]. Coleopterans in particular use a range of defences including defecation and reflex bleeding [52]. When the beetle bites vertebrates, it does not solely inflict physical pain onto the handler, but also releases chemical substances.

Gyrinids, for instance, produce, among other substances, norsesquiterpenes [24,53,54]. Norsesquiterpenes have been established to be toxic to fish [54,55]. The literature on either the pharmacological effects or toxicity of norsesquiterpenes to humans is relatively patchy. However, some norsesquiterpenes, like ptaquiloside (produced by certain ferns), have been suspected to be carcinogenic to humans [56]. Dytiscids on the other hand also possess prothoracic defensive glands, which produce, among other substances, hormone-like steroids [24].

It entailed delicate consideration to determine whether the target beetles’ release of defensive compounds was a consumptive or non-consumptive interaction. To draw inferences from predator–prey interactions [35], we posit that a predator–prey interaction is considered consumptive if the predator kills the prey. However, if the prey releases defences in response to the perceived risk from the attacker, the predator avoidance reaction is not considered consumptive [37,57]. In terms of human–animal interactions, ref. [27] states that consumptive use does not necessarily need to lead to mortality, but rather, the extraction of derivatives qualifies the use as consumptive. Therefore, if the intent is to capture and allow the organism to bite and release its venom or digestive enzymes, and upon being captured, the organism does release, then we can confidently categorise these uses as comprising elements of consumption.

We maintain this classification because the literature indicates that certain defence compounds and enzymes are also required for digestion purposes. In this case, refs. [58,59] note that many predaceous insects use their venom to escape predation (including human handlers). While it is possible to capture and release beetles back into their natural habitat without harming them, this raises concerns regarding the amount of venom the insect injects into a human handler relative to the amount required for defence against non-human predators and the amount of venom or digestive enzymes required for prey digestion. This is an essential consideration because insect chemical defences are not infinite; they can be depleted [50,52]. If we consider the released defence mechanisms (from the beetles to the users) to be derivatives (venom), it seems reasonable to categorise the ‘use’ component of this group of rituals as consisting of characteristics of consumptive use, or specifically as representing sub-lethal effects on the beetles.

One of the key determinants of whether a use of a biotic resource is consumptive or non-consumptive is whether the resource is removed from its natural environment [27,28,30]. None of the uses in this category lead to mortality. A critical consideration is whether the rituals are performed in situ (at the water source) or ex situ (varying distances from the water beetle’s natural habitat, e.g., home) and whether, after use, they are released back to their natural environment. If the ritual is performed in situ, we can easily assume that the beetles would crawl back into the water. We gathered from the study that most of the water beetle rituals are performed with adult beetles as opposed to their immature counterparts; see, for example, [18]. This is an essential consideration, because it is critical to understanding the implications of in situ use versus ex situ release of the beetles. When compared to their immature counterparts, most adult water beetles are capable of flying, as they regularly fly over water and over land whenever necessary [60].

In most cases, water beetles typically enter and leave a water body in search of mates or if water conditions are not optimal for their survival [61]. This is in part due to the fact that most adult aquatic and semi-aquatic coleopterans families are air breathers (atmospheric air breathing) rather than gill breathers (dissolved oxygen). Some of the documented atmospheric air-breathing aquatic coleopterans include Dytiscidae, Elmidae/Drypidae, Gyrinidae, Haliplidae, Hydraenidae, Hydrophilidae, Curculionidae; see, for example, [21,22]. Therefore, we can infer that if the rituals are performed and the beetles are released offsite, the used beetles will be able to locate a conducive water body nearby.

The emphasis on distance is critical for two reasons. First, the removal of water beetles from water may lead to desiccation or dehydration of the specimens. While beetles are said to be one of the best adapted arthropods to desiccation [62], we cannot be entirely certain of the specific lengths of time they can withstand dehydration and the conditions under which they are kept during the ritual. Second, we believe that the assumption about the majority of water beetles’ ability to fly is overly broad and hence deserves qualification. In this instance, we emphasise that flight abilities exhibit significant variation between different species. According to [63], several Gyrinidae species may fly for up to 20 km. However, it is important to point out that some species within the same family have limited flying capabilities, while other species are completely devoid of the ability to fly. In a comparable instance, the Dytiscidae family displays considerable variability in terms of flying abilities exhibited by different species. Notably, many species within this family possess flight capacities that encompass a spectrum ranging from less than 1 kilometre to distances beyond 20 km [64]. Regardless of these factors, the presumption that the beetles will return to their natural environment after use is not certain. First, if the ritual is performed away from the site, the distances would have to be within the beetles’ flight abilities; second, the beetles must not have been injured during capture, usage, or release, which could potentially impair mobility; and third, the beetles must survive the threat of predation by terrestrial predators as they make their way towards a nearby waterbody. If the beetles are removed and are unable to return to their habitat for one of these three reasons, or for any other reason, the handling and execution of the rituals offsite could have a negative impact on the population densities of the beetles.

A category of uses that may be closer to this group of uses is recreational fishing. Neither use leads to mortality, alterations, or permanent removal of the organism from its natural habitat. Recreational fishing can either be non-consumptive or consumptive. According to [65,66], recreational fishing is considered consumptive if it involves the exploitation of fish stocks. It is considered non-consumptive if the fish are merely caught and then released (catch and release) [66]. The catch-and-release interaction differs from this group of uses (catch–use–release) because the recreational fishing counterpart does not involve the “use” phase of the organism. However, to classify catch and release as non-consumptive, one risks overlooking salient considerations. These include potential sub-lethal physiological disturbances experienced by the animals during the invasion of their natural environment and the implications of the display of risk-avoidance behaviour on the animal’s fitness and reproduction. A further consideration is that all of these may impact the animal’s stocks, migration patterns, and the resultant demographics (see, for example, arguments by [44,45]).

### 4.4. Uses of Water Beetles Resulting in Mortality

We regarded the last group of rituals as both lethal (consumptive) and destructive uses of water beetles. Mortality or lethality is the most prominent determinant of the consumptive use of animals [27,30,32,35] and destructive human–animal interactions [48,67,68] widely documented. The literature suggests that certain families of beetles are consumed as a food source. The practice of eating insects, or entomophagy, is common. Global estimates suggest that approximately 2 billion people participate in entomophagy [69]. In addition, we observed in the literature that water beetles are ingested by humans as part of traditional medical interventions.

In Madagascar, for instance, we gathered that dytiscids in particular are cooked and eaten both as food and as a cough prevention remedy (Decary [70], cited in [18]). The ingestion of water beetles (e.g., gyrinids) is, however, not exclusively practised in SSA. A review by Jach and Balke [71] suggests that gyrinids in particular play a prominent role in traditional cuisines and medicines and have weighty cultural importance to many indigenous communities across the globe. For instance, ref. [72] and Ochs [73] (cited in Jach and Balke [71]) states that in the early 19th century, *Aulonogyrus strigosus F*. (Gyrinidae), was roasted and consumed by Australian aboriginal communities. At approximately the same time, gyrinids were used in Europe as an aphrodisiac for cows and mares [71].

We did not expect that humans could eat water beetles. This is because water beetles, like gyrinids, exude a strong-smelling secretion from their pygidial glands as part of their defence response to predation [74,75,76,77]. These secretions have been previously found to repel fish predators, based on a bioassessment conducted by [74], where gyrinids were fed to fish and, after a number of trials, it was observed that the fish rejected the beetles on sight. From these tests, it was concluded that gyrinids were unpalatable to predators. Hence, most predators tend to avoid these beetles ([76], cited in [51]).

Do all lethal interactions, or interactions leading to mortality, equate to consumptive use? This remains disputed. Ref. [33]’s perspective of the consumptive use of animal species posits that lethal uses become concerning if they negatively affect threatened species. With respect to most invertebrate species, determining whether particular species are threatened is difficult. In the majority of instances, ascertaining the vulnerable status of specific invertebrate species presents inherent difficulties. One component of this data challenge is that data on hyperdiverse or megadiverse species (i.e., beetles) is lacking [78]. On the other hand, this could be ascribed to observed biases in the IUCN Red List data for threatened species, which currently tend to favour vertebrates over invertebrates [79,80]. Studies have indicated acute institutional biases against invertebrates (institutional vertebratism) [81,82] and taxonomic chauvinism [81]. These biases result in a failure to recognise that, according to estimates, more than 70 invertebrate species have become extinct over the last 600 years [83]. Such prejudices could be detrimental to global conservation efforts for invertebrates (particularly insects). Biodiversity data plays a crucial role in facilitating the sustainable use of animal resources by providing a precautionary management reference for determining appropriate rates and timing of harvesting activities [84].

## 5. Conclusions

In this study, we classified traditional uses of water beetles using the dichotomous concepts of “consumptive” versus “non-consumptive” uses. The concepts informed the determination of the extent to which traditional uses of water beetles could be considered threatening to species biodiversity and abundance. In the classification, we recognise the difficulty imposed by the inexplicit nature of the application of the concepts when classifying the uses of wildlife and animal resources. From the study we concluded that caution should be exercised when categorising human–animal interactions using these concepts. The observation made indicates that the process of classification, when conducted meticulously, requires a significant amount of data. It cannot be simply handled without careful consideration of taxa-specific sensitivities or tolerance to human interactions. The classification process, in our case, was conducted without access to region-specific data regarding the conservation status, population size, seasonal population fluctuations, reproductive rates, and physiological implications of the interactions for these organisms. As a result, our assessment of the majority of the interactions remained inexhaustive. Nonetheless, the classification used in this study enabled us to conclude that the utilisation of water beetles for the fulfilment of traditional and medical requirements in most parts of SSA is seldom lethal and has minimal direct impact on the population sizes of water beetles.

## Figures and Tables

**Table 1:** Traditional African rituals performed using whirligig beetles (gathered through focused group discussions conducted in KwaZulu Natal province, South Africa, for the current study).

Use of the Beetle	Taxa: Specificity	Implications for Demographics
Breast growth stimulant	Gyrinidae	Non-lethal use: catch, use, and release (after the ritual, the beetle is released)
Use of the beetle for unlocking oral whistling	Gyrinidae	Non-lethal use: catch, hold, and allow the beetle to bite the tip of the tongue and release (released alive).
Use of the beetle in learning how to swim or to improve swimming abilities	Gyrinidae and water beetles (non-specific)	Non-lethal use: catch, hold, and allow the beetle to bite the target body part.

**Table 2:** Traditional African rituals performed using water beetles gathered from the current study and from the consulted literature.

Use of the Beetle	Taxa: Specificity	Where Ritual Is Performed	Implications for Demographics	Source
Sorcery (symbol of curse)	Gyrinidae	Tanzania	Use in metaphors	[24]
Proverb: teaching people to be responsible for their wellbeing	Dytiscidae	Madagascar	Use in metaphors	[18,39]
Fish presence indicator (when there are water beetles, it is a sign that there will be fish	Dytiscidae	Tanzania and Burundi	Non-lethal use: intangible use. The presence/absence of the beetle is used as a biological indicator of the presence of fish	[18]
Indicator of water of good quality, clear water	Dytiscidae	Tanzania (Chaga)	Non-lethal use, intangible use	[18]
Breast growth stimulant	Water Beetles (non-specific), Gyrinidae and Dystiscidae	Uganda, Kenya, Congo, Cameroon, Kenya, Rwanda, Zimbabwe	Non-lethal use: catch, use, and release (after the ritual, the beetle is released)	[18,24,40]
Use of the beetle for unlocking oral whistling	Gyrinidae	Zimbabwe	Non-lethal use: catch, hold, and allow the beetle to bite the tip of the tongue and release (released alive)	[24,41]
Use of the beetle in learning how to swim	Water beetles (non-specific)	Zambia and Madagascar	Non-lethal use: catch, hold, and allow the beetle to bite the target body part	[18,24]
Treatment of gynecomastia	Gyrinidae and Dytiscidae	Tanzania	Non-lethal use: catch, hold, and allow the beetle to bite the target body part and release	[24]
Treatment of traditional circumcision wounds	Gryrinidae	South Africa	Non-lethal use: catch, hold, and allow the beetle to bite the target body part (released alive)	[40]
Used for the treatment of dizziness	Water beetles (non-specific)	Tanzania	Non-lethal use: catch, use, and release (alive)	[24]
Water purification	Dytiscidae	Tanzania	Non-lethal use: involves catch, use, and release	[18]
Prevention of cough	Dytiscidae	Madagascar	Lethal use: catch, cook and eat the beetle	[18]
Improvements in swimming abilities	Water beetles (non-specific)	Zimbabwe	Lethal use: catch and eat the beetle	[18]
Eaten as a source of food	Gyrinidae and Dytiscidae	Central African Republic, Benin, Senegal, Sierra Leone, Congo, Madagascar, and Togo	Lethal use: caught, prepared, and eaten	[18,25,26]

## Data Availability

The data presented in this study are available on reasonable request from the corresponding author. The data are not publicly available due to privacy reasons.
